# Carbuncle, Local Treatment

**Published:** 1874-09

**Authors:** Peter Eade


Local Treatment of Carbuncle.—*By Peter Eade, M.D.*—In a short communication published in *The Lancet* of 1870, I recorded the particulars of a case of extensive carbuncle of the back of the neck, which I had treated by the free application of carbolic acid to the diseased part, and especially by its free insertion into the holes and sinuses which had formed, as is usually the case, over the central portion very early in the course of the disease. An experience since that time of two or three cases of large carbuncle, and of many cases of carbuncular boils, treated by the same method, has proved to me that the action of this remedy is so definite and so constantly beneficial that I have no hesitation in recommending it to the favorable notice of the profession. * * * The strength of the solution of carbolic acid which I have employed has been about one part of the acid to four or five of the solvent, (oil or glycerine) and its efficacy, I would repeat, has appeared to be limited almost absolutely to those parts with which it could be brought into actual contact; and although it appears occasionally to have produced injurious effects when used in large quantity; yet I have kept a large sloughing and granulating surface for days together constantly covered with the carbolised oil, without any harm arising, although the urine soon presented the peculiar blackish color which has been several times observed during its employment.—*The London Lancet—June.*

